# The Role of *ONECUT1* Variants in Monogenic and Type 2 Diabetes Mellitus

**DOI:** 10.2337/db23-0498

**Published:** 2023-11-01

**Authors:** James Russ-Silsby, Kashyap A. Patel, Thomas W. Laver, Gareth Hawkes, Matthew B. Johnson, Matthew N. Wakeling, Prashant P. Patil, Andrew T. Hattersley, Sarah E. Flanagan, Michael N. Weedon, Elisa De Franco

**Affiliations:** 1 Department of Clinical and Biomedical Sciences, University of Exeter Faculty of Health and Life Sciences, Exeter, United Kingdom; 2 The Society for the Rehabilitation of Crippled Children Narayana Health Children’s Hospital, Mumbai, India

**Keywords:** Neonatal Diabetes, Type 2 Diabetes, Maturity-Onset Diabetes of the Young (MODY), Genetic Research, Monogenic Diabetes, Clinical Genetics, Pancreatic Development

## Abstract

ONECUT1 (also known as HNF6) is a transcription factor involved in pancreatic development and beta-cell function. Recently, biallelic variants in *ONECUT1* were reported as a cause of neonatal diabetes mellitus (NDM) in 2 subjects and missense monoallelic variants were associated with type 2 diabetes and possibly maturity-onset diabetes of the young (MODY). Here we examine the role of *ONECUT1* variants in NDM, MODY and Type 2 diabetes in large international cohorts of subjects with monogenic diabetes and >400,000 subjects from UK Biobank. We identified a biallelic frameshift *ONECUT1* variant as the cause of NDM in one individual. However, we found no enrichment of missense or null *ONECUT1* variants among 484 individuals clinically suspected of MODY, in whom all known genes had been excluded. Finally, using a rare variant burden test in the UK Biobank European cohort, we identified a significant association between heterozygous *ONECUT1* null variants and type 2 diabetes (*P=*0.006) but did not find association between missense variants and type 2 diabetes. Our results confirm biallelic *ONECUT1* variants as a cause of NDM and highlight monoallelic null variants as a risk factor for type 2 diabetes. These findings confirm the critical role of *ONECUT1* in human beta-cell function.

## Graphical abstract

**Figure:**
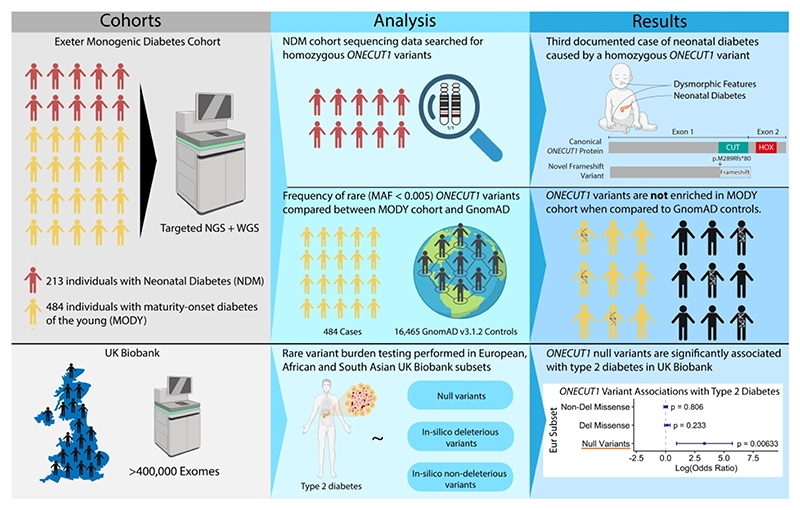

## Introduction

Pancreatic transcription factors mediate the process of cellular differentiation from pluripotent stem cells through to mature pancreatic cells. Genetic variants that disrupt pancreatic transcription factors’ function result in a wide spectrum of pancreatic phenotypes, ranging from diabetes in the first six months of life (neonatal diabetes, NDM) to increased risk for type 2 diabetes (1–3). Pathogenic variants in a subset of pancreatic transcription factors are common causes of maturity-onset diabetes of the young (MODY), with variants affecting the hepatocyte nuclear factor (HNF) genes *HNF1A, HNF1B* and *HNF4A* accounting for more than 50% of cases of the disease (4).

The latest HNF gene to have been shown to have a role in monogenic diabetes is *ONECUT1* (*HNF6*), with two unrelated cases of NDM caused by biallelic *ONECUT1* pathogenic variants reported (5). These individuals, both of whom had pancreatic hypoplasia, were homozygous for different coding variants in the *ONECUT1* gene; one missense (p.Glu231Asp), the other stop-gain (p.Glu231*). The authors also identified a significant enrichment of heterozygous rare *ONECUT1* variants (minor allele frequency [MAF] <0.005) in a cohort of 2165 individuals with type 2 diabetes when compared to healthy controls, suggesting a role of *ONECUT1* in adult-onset diabetes. Furthermore, the relatively young age profile and low BMI of *ONECUT1* variant carriers in the type 2 diabetes cohort was consistent with a role of certain pathogenic *ONECUT1* variants in autosomal dominant monogenic diabetes (5).

*ONECUT1* has long been considered a candidate gene for monogenic diabetes, as it plays a vital role in pancreatic endoderm and pancreatic progenitor cell differentiation (6). Through co-operative binding with other pancreatic transcription factors, including PDX1, GATA6, and FOXA2, it regulates gene expression during the early stages of pancreatic development and facilitates the maturation of precursor cells into functional adult pancreatic cell types. In addition to this early developmental role, ONECUT1 is also important for development and maintenance of beta-cell identity (6).

In this study, we build upon recent findings by further exploring the role of *ONECUT1* variants in monogenic and type 2 diabetes using a large cohort of individuals with suspected monogenic beta-cell disorders and >400,000 individuals from UK Biobank (7).

## Research Design and Methods

### Participants in the Exeter monogenic diabetes cohort

We studied an international cohort of 697 subjects referred to the Exeter Genomics Laboratory for monogenic diabetes testing, in whom all the known genetic causes of monogenic diabetes had been excluded as previously described (8). 213 unrelated individuals were diagnosed with NDM (defined as diabetes with onset <6 months old) and 484 unrelated individuals were clinically suspected of having MODY.

Whole-Genome Sequencing (WGS) was performed on 333 individuals (172 NDM, 161 MODY) as previously described (9), with the remaining 323 individuals suspected of MODY and 41 individuals with NDM tested on our targeted next generation sequencing panel (10), which had been updated to include baits for *ONECUT1*. The study was conducted in accordance with the Declaration of Helsinki, and all subjects or their parents gave informed consent for genetic testing with ethical approval received from the Genetic Beta Cell Research Bank, Exeter, UK.

### Exploring the role of biallelic *ONECUT1* variants in NDM

We interrogated the sequencing data of the 231 individuals with NDM without a pathogenic variant in a known gene to identify homozygous or compound heterozygous *ONECUT1* pathogenic variants. Only biallelic coding or splice site variants (+/-2bp of intron/exon boundary) with a MAF<0.0001 in gnomAD v2.1.1 (11) affecting the canonical transcript (NM_004498) were considered as potentially pathogenic.

### Examining the role of monoallelic *ONECUT1* variants in MODY

We first analysed the sequencing data of 484 individuals clinically suspected of MODY without a genetic diagnosis to detect any null (here defined as frameshift, stop-gain, start-loss, canonical splice site variants and single/multi exon deletions) *ONECUT1* variant affecting the canonical transcript. We performed Fisher’s exact tests to investigate enrichment of *ONECUT1* variants in this cohort when compared to 16,465 individuals included in the gnomAD v3.1.2 control population (11). For the analysis to be comparable to that conducted by Philippi *et al*. (5), which detected an enrichment of missense variants in the young-onset type 2 diabetes cohort, the same gnomAD v2.1.1 MAF cut-off (0.005) was used to filter variants in the case and control cohorts. We investigated synonymous variant enrichment in both case and control cohorts, which acted as a negative control to ensure that any differences in the sequencing technologies used did not influence variant frequency.

### Analysis of UK Biobank data to examine the association between heterozygous *ONECUT1* variants and Type 2 diabetes

We performed burden testing for association between type 2 diabetes and *ONECUT1* variants using the UK Biobank exomes dataset (7). Separately, we tested the European (n=419,850, of which 32,899 had type 2 diabetes), African (n=7,393, of which 1,356 had type 2 diabetes), and South Asian (n=9,539, of which 2,431 had type 2 diabetes) UK Biobank cohorts. The definition of type 2 diabetes used here was a report of non-insulin dependent diabetes from any source or a HbA1c level >= 48mmol/mol. We excluded individuals receiving insulin or with an additional conflicting diagnosis of type 1 diabetes.

We created three subsets of *ONECUT1* variants; null variants (as previously defined in this study), *in silico*-predicted deleterious missense variants (CADD phred score >=25) and non-deleterious missense variants (CADD phred score <25). Only variants with a MAF <0.001 that affect the canonical *ONECUT1* transcript were included in the subsets.

The variant subsets were tested for association with type 2 diabetes using the UK Biobank analysis pipeline developed by Regeneron (12). Due to the lack of *ONECUT1* null variants in the African and South Asian cohorts, the null subset could only be tested in the European cohort. The two missense variant subsets were tested in all three cohorts. Age, sex, testing centre, exome batch and principal components 1-40 were all included as control variables. When testing for association with binary traits including type 2 diabetes, the pipeline performs a Firth-corrected logistic regression statistical test.

## Results

### Identification of the third NDM case caused by a biallelic *ONECUT1* variant

**Figure 1:**
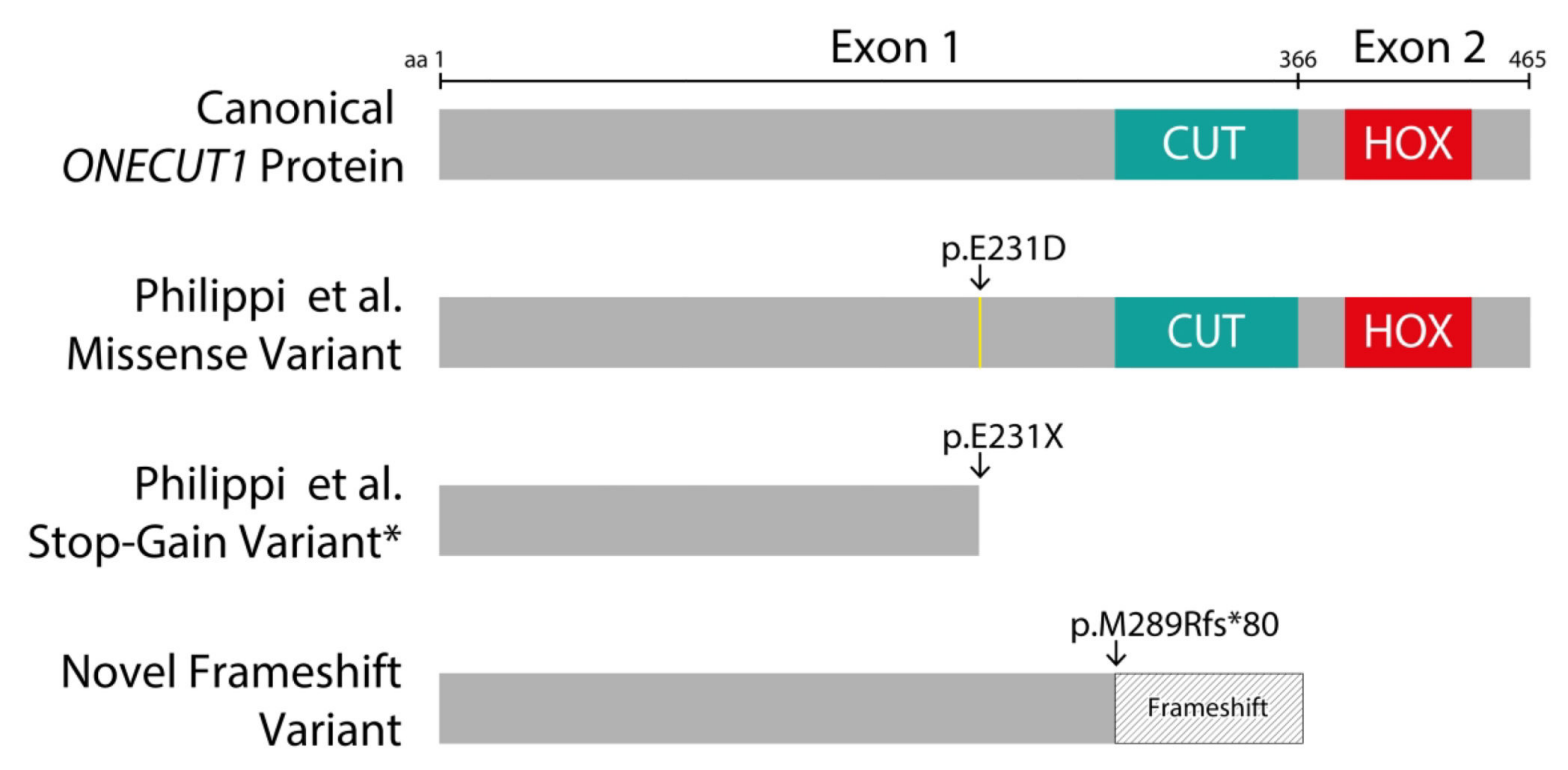
Comparison of the predicted effect of the variants identified in the three neonatal diabetes cases reported to date on the ONECUT1 protein. Both null variants result in the complete loss of the CUT and homeobox domains of the protein, possibly explaining the more severe phenotypes observed in the two cases. *As the Phillippi et al. stop gain variant causes a termination of translation ahead of the splice junction between exons 1 and 2, it could result in complete loss of protein through nonsense mediated decay.

Within our NDM cohort of 231 individuals, we identified one novel biallelic variant in the *ONECUT1* gene. The homozygous frameshift variant (p.Met289Argfs*80, Chr15(GRCh37):g.53081217_53081218dup) was detected in a single proband referred from India. The p.Met289Argfs*80 variant is predicted to result in the translation of a truncated ONECUT1 protein lacking the CUT and HOX domains (Figure 1).

The female proband presented with intra-uterine growth restriction (birth weight was 1590g at 40 weeks gestation; -6.42 SD) and was diagnosed with diabetes at three days of age. She was treated with insulin (0.15-0.2 U/kg/day) from diagnosis but displayed postnatal growth failure and died at 2 months of age. Exocrine pancreatic function was not tested. There was no known family history of diabetes. In addition to diabetes, the proband had multiple extra-pancreatic features including facial dysmorphism, skeletal dysplasia, camptodactyly, epilepsy, hypotonia and anaemia.

### *ONECUT1* variants are not enriched in the Exeter MODY cohort when compared with gnomAD controls

*ONECUT1* missense variants were not significantly enriched in our cohort of 484 individuals with suspected MODY when compared to population controls (Fisher’s exact *P* = 0.644). There were 9 individuals in the Exeter MODY cohort with coding variants in *ONECUT1* (all heterozygous missense, listed in supplementary table 1), compared to 384 individuals in the gnomAD control cohort (381 heterozygous, 3 homozygous). There were no null variants detected among the suspected MODY cases (10 individuals with heterozygous null variants were present in the controls). No significant difference in the frequency of synonymous variants was found between the MODY and gnomAD cohorts (Fisher’s exact *P* = 0.822), confirming that the cohorts are reliably comparable and not significantly affected by technical differences in sequencing platforms.

### Null variants are significantly associated with type 2 diabetes in UK Biobank

**Figure 2:**
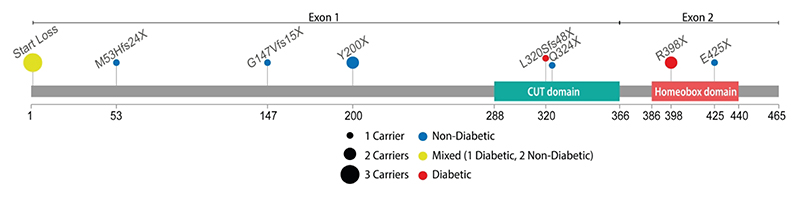
Protein positions of the 12 heterozygous *ONECUT1* null variants observed in the UK Biobank and the type 2 diabetes status of the individuals who carry them. Three of the four diabetic individuals carry variants within the CUT and Homeobox protein domains.

We identified 12 individuals heterozygous for *ONECUT1* null variants in UK Biobank (Figure 2). Four of these cases (33%) had Type 2 diabetes, with a median age of diagnosis of 58 years (IQR = 57.5-63.5), and BMI of 30.4 (IQR = 29.1-33.3). A significant association was found between null variants and type 2 diabetes in the UK Biobank European cohort (OR=27.90, 95% CI=2.56-304.35, *P =* 0.00633). The South Asian and African cohorts were not tested in the null variant analysis due to the lack of null variants in these UK Biobank subsets.

No association with type 2 diabetes was found for either CADD-predicted deleterious missense variants or CADD-predicted non-deleterious missense variants in the European, African or South Asian Cohorts (Table 1). There were 9 individuals (3 in the European Cohort and 6 in the African cohort) homozygous for *ONECUT1* missense variants in the UK Biobank (1/9 had a CADD-predicted deleterious variant); none of these individuals had a clinical diagnosis of diabetes or an HBA1c>48mmol/l.

## Discussion

In this study, we explored the role of *ONECUT1* variants in the aetiology of monogenic and type 2 diabetes. Our assessment of 697 individuals referred for NDM or MODY testing identified a third case of neonatal diabetes caused by a homozygous *ONECUT1* variant. Furthermore, using UK Biobank, we detected an association between heterozygous null *ONECUT1* variants and increased risk of adult-onset diabetes.

With the report of the third case of NDM caused by a homozygous *ONECUT1* variant, the requirement of three unrelated cases to confirm a gene-disease association, as is required by Genomics England for a gene to be added to their disease panels, has now been met (13). As a result, we recommend that *ONECUT1* is added to gene panels for NDM.

The identification of a third NDM case provides additional details on the phenotype associated with this rare NDM subtype. The previously described individual (5) homozygous for the *ONECUT1* missense variant had a relatively milder phenotype (diabetes onset after 6 months of age and no extra-pancreatic features) when compared to both the previously described individual with the homozygous stop-gain variant, and the individual in our cohort with a homozygous frameshift variant. This could possibly indicate that some function is preserved with the missense variant, while there is complete loss with homozygous null variants. Both individuals with homozygous null variants had extremely low birthweights (both <-5SD), suggesting reduced insulin secretion *in utero*, similarly to what has been reported in other forms of NDM characterised by absence of fetal insulin (14). The birthweight of the individual with the homozygous missense variant was less extreme at -1.4SD. Both individuals with homozygous null variants had additional morphological and neurological features, while the phenotype of the individual with the homozygous missense variant was limited to pancreatic hypoplasia and NDM. The presence of extra-pancreatic features is consistent with the phenotype of mouse knockout models, which display impaired development of liver, gallbladder and pancreas (Table 2) (15–19). The possibility of some retained function in the missense variant protein is supported by the fact that unlike the two null variants, the missense variant does not directly disrupt the known functional domains of the ONECUT1 protein. These observations are suggestive of a possible genotype-phenotype relationship in *ONECUT1*-NDM, with null variants causing a multi-system syndromic disease whilst variants which do not result in complete loss of protein function cause a pancreatic-only phenotype. The identification of additional individuals with biallelic pathogenic *ONECUT1* variants is needed to further define the clinical features of this rare NDM subtype.

We show that null variants in *ONECUT1* are likely to be a risk factor contributing to the development of type 2 diabetes in adulthood. The odds ratio of the observed UK Biobank association is high compared to other genes where rare variants have been associated with type 2 diabetes, such as *GIGYF1*, in which null variants were associated with type 2 diabetes with an odds ratio of 4.5 (CI = 2.71-6.37) (20). The magnitude of the association is in fact similar to that observed for heterozygous null *RFX6* variants (21), which are associated with MODY with reduced penetrance. However, the small number of variants (n=9) and wide confidence interval of the association between *ONECUT1* null variants and type 2 diabetes mean that caution must be taken in the interpretation of the result.

Screening in our large cohort of individuals with clinically suspected MODY did not reveal an enrichment of *ONECUT1* missense variants and no individuals within the cohort had a null variant in the gene. Similarly, Prudente *et al*. (22) did not find any predicted pathogenic *ONECUT1* variants in their cohort of familial diabetes cases. Taken together this suggests that monoallelic variants in *ONECUT1* are either not a cause of monogenic diabetes or they represent an extremely rare subtype of the disease. Additional studies of larger MODY cohorts are needed to determine the role of *ONECUT1* variants in monogenic adult-onset diabetes.

In summary, our study provides further evidence showing the role of biallelic *ONECUT1* variants in NDM. Furthermore, we identified a possible role for heterozygous *ONECUT1* null variants as a risk factor for adult-onset type 2 diabetes. Our results further define the role of *ONECUT1* variants in both neonatal and adult-onset diabetes, confirming the important role of this transcription factor in beta-cell development and function.

## Supplementary Material

Supplementary Materials

## Figures and Tables

**Table 1:** Results of burden testing analysis in UK Biobank. The only significant association identified was between null variants and type 2 diabetes in the UK Biobank European cohort. Due to the lack of *ONECUT1* null variants in the African and South Asian Cohorts, they could only be tested in the European cohort.

Variant Subset	N Individuals with *ONECUT1 *Variants	OR (95%CI)	*P*
European (N=419,850)			
Null Variants (N=9)	12	27.898 (2.557-304.345)	0.00633**
In-Silico Predicted Deleterious Missense Variants (N=99)	1,012	1.158 (0.915-1.466)	0.233
In-Silico Predicted Non-Deleterious Missense Variants (N=194)	1,915	1.021 (0.863-1.184)	0.806
African (N=7,393)			
Null Variants (N=0)	0	NA	NA
In-Silico Predicted Deleterious Missense Variants (N=8)	20	0.633 (0.199-2.015)	0.439
In-Silico Predicted Non-Deleterious Missense Variants (N=14)	286	0.437 (0.115-1.662)	0.225
South Asian (N=9,539)			
Null Variants (N=0)	0	NA	NA
In-Silico Predicted Deleterious Missense (N=11)	29	0.580 (0.230-1.450)	0.244
In-Silico Predicted Non-Deleterious Missense Variants (N=25)	49	0.712 (0.347-1.462)	0.355

**Table 2:** Phenotypic summary of all individuals found to have pathogenic biallelic variants in the *ONECUT1* gene. Details of the phenotype observed in *Onecut1* knockout mouse models is also provided for comparison. In the NDM case we identified some clinical features may have been missed due to limited access to the patient and their death at two months. The mouse phenotypes were collated from previous studies looking at the effect of *Onecut1* knockouts (15–19).

	Glu231Asp Homozygote (Philippi et al.)	Glu231* Homozygote (Philippi et al.)	Mer289Argfs* 80 Homozygote (this report)	Mouse Biallelic *Onecut1* Knockout
Pancreatic Phenotypes	Pancreatic hypoplasia, Neonatal Diabetes	Pancreatic hypoplasia, Neonatal Diabetes	Neonatal Diabetes	Pancreatic hypoplasia, abnormal pancreas development, abnormal glucose homeostasis, absent pancreatic alpha and beta-cells, decreased delta cell number, disorganized pancreatic islets
Hepato-biliary Phenotypes	None identified	hepatomegaly, hepato-cellular insufficiency, jaundice, absent gallbladder	None identified	Abnormal hepatoblast differentiation, jaundice, absent gallbladder, biliary cysts
Retina Phenotypes	None identified	None identified	None identified	Retina outer nuclear layer degeneration, abnormal retina outer plexiform layer morphology
Musculoskeletal Phenotypes	None identified	Camptodactyly, hypotonia, microretrognat hism, lack of extension at elbow, prominent heel bone, convexly rounded sole	Camptodactyly, hypotonia, triangular face, microcephaly	None identified

## Article Highlights

- We confirmed homozygous *ONECUT1* variants as causative for neonatal diabetes with the identification of a third case.
- Rare heterozygous *ONECUT1* variants were not enriched in a cohort of 484 individuals clinically suspected of having maturity-onset diabetes of the young.
- Heterozygous *ONECUT1* variants are significantly associated with type 2 diabetes in the UK Biobank European population.
- No association was observed between heterozygous *ONECUT1* missense variants and type 2 diabetes in the UK Biobank.
