# Supplementary material for: The Role of ONECUT1 Variants in Monogenic and Type 2 Diabetes Mellitus
Source: Diabetes. Author manuscript; Available in PMC 2026 Jun 11. (PMC7619148; doi:10.2337/db23-0498)
Supplement: Supplementary Materials [file EMS213990-supplement-Supplementary_Materials.pdf]

Online Supplemental Material

Nucleotide Change	AA Change	Gnomad v2.1.1 AF	N Heterozygotes in Exeter MODY Cohort
Chr15(GRCh37):g.53049960C>T	p.Arg397His	0.0000199	1
Chr15(GRCh37):g.53081205T>C	p.Asn293Asp	0	1
Chr15(GRCh37):g.53081328G>A	p.Pro252Ser	0.0000125	2
Chr15(GRCh37):g.53081357A>G	p.Val242Ala	0.00112	1
Chr15(GRCh37):g.53081421G>C	p.His221Asp	0	1
Chr15(GRCh37):g.53081983G>T	p.His33Gln	0.00104	1
Chr15(GRCh37):g.53082004G>C	p.Asp26Glu	0.00131	1
Chr15(GRCh37):g.53082063T>C	p.Met7Val	0.0000400	1

Supplemental Table 1: Heterozygous *ONECUT1* Missense Variants identified in the Exeter MODY Cohort.
Eight different missense variants were identified in 9 individuals among a cohort of 484 individuals clinically suspected as having maturity-onset diabetes of the young. In all cases genetic samples were only available for the proband, therefore co-segregation studies could not be performed.

Downloaded from http://diabetesjournals.org/diabetes/article-pdf/doi/10.2337/db23-0498/733454/db230498.pdf by UNIVERSITY OF EXETER user on 29 August 2023
